# Suppressing Src-Mediated EGFR Signaling by Sustained Calcium Supply Targeting Triple-Negative Breast Cancer

**DOI:** 10.3390/ijms241713291

**Published:** 2023-08-27

**Authors:** Keun-Yeong Jeong, Seon Young Park, Min Hee Park, Hwan Mook Kim

**Affiliations:** 1 Gachon Institute of Pharmaceutical Science, Gachon University, Incheon 21936, Republic of Korea; audijeong@naver.com (S.Y.P.); pmh1880@hanmail.net (M.H.P.); 2 MetiMedi Pharmaceuticals Co., 40, Imi-ro, Uiwang-si 16006, Republic of Korea

**Keywords:** breast cancer, triple-negative, lactate calcium salt, Src, EGFR

## Abstract

Src is emerging as a promising target in triple-negative breast cancer (TNBC) treatment because it activates survival signaling linked to the epidermal growth factor receptor. In this study, the effect of calcium supply on Src degradation was investigated to confirm underlying mechanisms and anticancer effects targeting TNBC. MDA-MB-231 cells, the TNBC cell line, were used. Calcium supply was feasible through lactate calcium salt (CaLac), and the applicable calcium concentration was decided by changes in the viability with different doses of CaLac. Expression of signaling molecules mediated by calcium-dependent Src degradation was observed by Western blot analysis and immunocytochemistry, and the recovery of the signaling molecules was confirmed following calpeptin treatment. The anticancer effect was investigated in the xenograft animal model. Significant suppression of Src was induced by calcium supply, followed by a successive decrease in the expression of epithelial growth factor receptor, RAS, extracellular signal-regulated kinase, and nuclear factor kappa B. Then, the suppression of cyclooxygenase-2 contributed to a significant deactivation of the prostaglandin E2 receptors. These results suggest that calcium supply has the potential to reduce the risk of TNBC. However, as this study is at an early stage to determine clinical applicability, close consideration is needed.

## 1. Introduction

Breast cancer is characterized by the presence or absence in the cells of three targetable markers, estrogen receptor (ER), progesterone receptor (PR), and/or human epidermal growth factor receptor 2 (HER2) [1]. Triple-negative breast cancer (TNBC) is defined as negative for ER/PR and HER2 amplification and accounts for 10–16% of all breast cancer cases [2]. The absence of these receptors means that there are fewer targeted medicines. Although neoadjuvant anthracycline-, platinum- and taxane-based chemotherapy are responded to well in early-stage TNBC, it has a big chance to develop the metastatic form that is virtually incurable due to drug resistance [3,4]. Especially, platinum-based chemotherapy has shown short-term efficacy in neoadjuvant chemotherapy, but it has not yet demonstrated an improved efficacy in advanced TNBC [4,5]. Of course, from an up-to-date therapeutic perspective, the past few years have witnessed remarkable progress in progression-free survival and overall survival in patients with TNBC through novel agents such as immunotherapy, antigen drug conjugates, and poly ADP-ribose polymerase inhibitors [6]. However, TNBC is still considered to be more aggressive and has a poorer prognosis compared with other types of breast cancer. In this background, the need for more diverse therapeutic options that can target metastatic TNBC could be emphasized. Epidermal growth factor receptor (EGFR) is commonly overexpressed in the vast majority of TNBCs, and there is considerable evidence linking crosstalk between EGFR and a proto-oncogene tyrosine-protein kinase, Src [7,8]. Src upregulates nuclear factor-κB (NF-κB) activity through signal transduction of the mitogen-activated protein kinase pathway [9,10]. Then, NF-κB combines with other signaling pathways to increase epithelialmesenchymal transition, angiogenesis, metastasis, stemness of cancer cells, and resistance to chemotherapy [2,10]. According to clinical analysis, the expression level of Src in TNBC is close to 90% without relation to tumor size, lymph node status, or lymphovascular invasion [11]. Therefore, Src is emerging as a promising target in TNBC treatment, and an Src kinase inhibitor, dasatinib, has been tried for TNBC treatment in clinical trials [12]. Our previous study demonstrated an anticancer effect on luminal A breast cancer by calcium-dependent Src degradation based on the background that estrogen binding to ER can promote rapid and transient interactions with cellular Src for activation of survival signaling [13]. Here, integrated with the aforementioned theory, given that survival signaling of any key receptors in a certain type of breast cancer could be mediated by Src irrespective of genetic association, it can be expected that calcium-dependent Src suppression would be shown to have a promising anticancer effect targeting TNBC as well. In this study, therefore, we investigated the inhibitory effect on Src and signaling changes related to survival threat in TNBC through sustained calcium supply.

## 2. Results

### 2.1. Sustained Calcium Supply Inhibits the Src-Dependent Trans-Activation of EGFR in TNBC Cells

**Figure 1:**
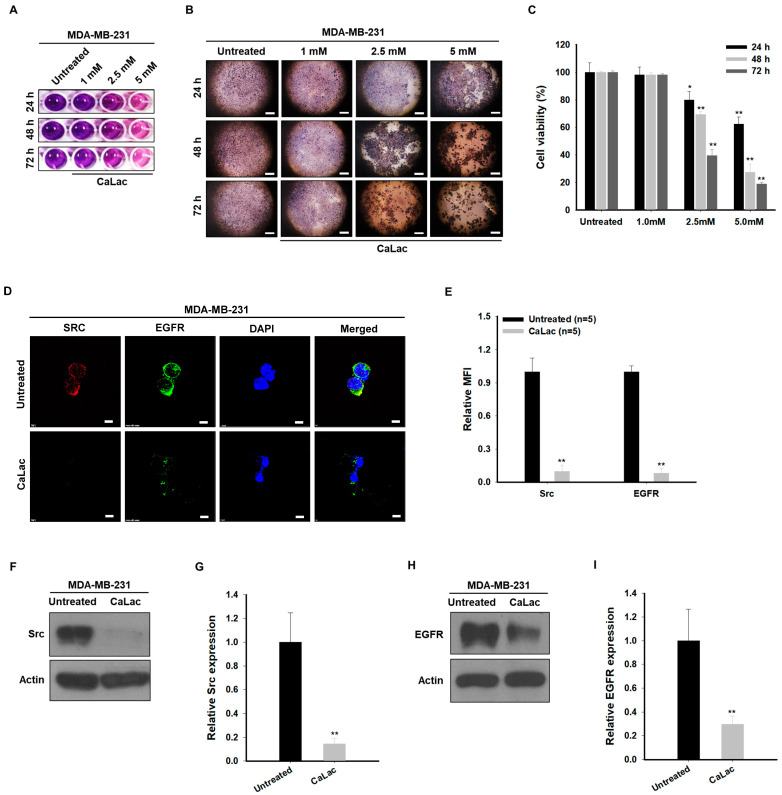
Investigation of optimal lactate calcium salt (CaLac) concentration for calcium supply and of the calcium effect on the expression of epithelial growth factor receptor (EGFR) and Src in triple-negative breast cancer cells. (**A**) Representative pictures for MTT assay following calcium supply with different concentrations (1, 2.5, or 5 mM). CaLac was used as a material for calcium supply. (**B**) Microphotographs from the MTT assay plate. Scale bars = 1 mm. (**C**) Quantitative analysis for the changes in cell viability according to calcium concentration and exposure time. The bar graphs were made based on triplicate analysis. (**D**) Immunocytochemical analysis for the expression of Src and EGFR following 5 mM CaLac supply for 48 h. Scale bars = 10 µm. (**E**) Quantitative analysis for immunocytochemical expression of Src and EGFR by mean fluorescence intensity (MFI). A total of 1 × 10^3^ cells was analyzed in each case. (**F**,**H**) Western blot analysis for Src or EGFR expression following 5 mM CaLacs supply for 48 h. The source films of Western blot data are provided in Supplementary Figure S1. (**G**,**I**) Quantitative analysis of the protein expression level of Src or EGFR. The bar graphs were made based on triplicate analysis. * *p* < 0.05 vs. Untreated; ** *p* < 0.001 vs. Untreated. Results are represented as means ± standard deviations.

The viability of TNBC was decreased gradually by increased calcium concentration (Figure 1A–C). There was no significant decrease in the viability with 1 mM calcium supply, however, a significant decrease in the viability was observed from 24 h following 2.5 and 5 mM calcium supply (Figure 1A–C). The rate of viable cells in the 48 h groups treated with 2.5 or 5 mM was about 69.5% and 27.4%, respectively, when the cell viability rate of the untreated group was calculated as 100% (Figure 1C). Based on the % of viability, a 5 mM calcium supply for 48 h was selected as a standard method to observe the changes in protein expression following the decrease in cell viability. A significant decrease in the expression of active forms of EGFR and Src in MDA-MB-231 cells was confirmed by the selected dose and time for calcium supply (Figure 1D–I). Fluorescence intensities of EGFR and Src were reduced by about 92% and 90.3%, respectively, compared with the untreated group (Figure 1D,E). The quantitative analysis for protein level indicated that the expression of the active form of EGFR and Src was reduced by about 70.3% and 85.4%, respectively, compared with the untreated group (Figure 1F–I).

### 2.2. Sustained Calcium Supply Inhibits the Sub-Signals Stimulated from EGFR and Src in TNBC Cells

**Figure 2:**
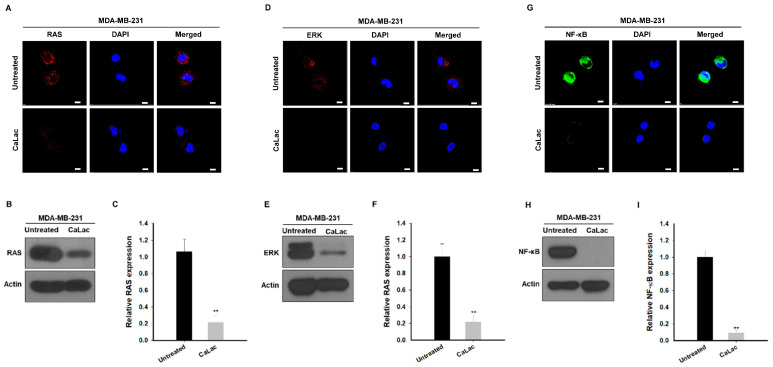
Inhibitory effect on sub-signaling of epidermal growth factor receptor (EGFR) and Src by calcium supply in triple-negative breast cancer cells. (**A**,**D**,**G**) Immunocytochemical analysis for the expression of RAS, ERK, and nuclear factor kappa B (NF-κB). Scale bars = 10 µm. (**B**,**E**,**H**) Western blot analysis for the expression of RAS, ERK, and NF-κB. The source films of Western blot data are provided in Supplementary Figure S2. (**C**,**F**,**I**) Quantitative analysis of the protein expression levels of RAS, ERK, and NF-κB. A quantity of 5 mM lactate calcium salt (CaLac) was supplied in the CaLac group for 48 h. The bar graphs were made based on triplicate analysis. ** *p* < 0.001 vs. Untreated. Results are represented as means ± standard deviations.

Changes in sub-signals depending on the decrease in the expression of EGFR and Src were investigated, and it was confirmed that there was an effect on the expression of signals leading from changes in RAS to ERK and NF-κB (Figure 2). The fluorescence images show that the intensity of RAS (red fluorescence), ERK (red fluorescence), and NF-κB (green fluorescence) tended to decrease qualitatively following calcium supply (Figure 2A,D,G). In quantitative analysis of protein expression, RAS was significantly reduced to about 79.5% (Figure 2B,C), ERK to about 78.5% (Figure 2E,F), and NF-κB to about 90% (Figure 2H,I) following calcium supply compared with the untreated group.

### 2.3. Calcium Supply Contributes to Inhibiting the Stimulation of Prostaglandin E2 Receptors (EP) Resulting from NF-κB Downregulation in TNBC Cells

**Figure 3:**
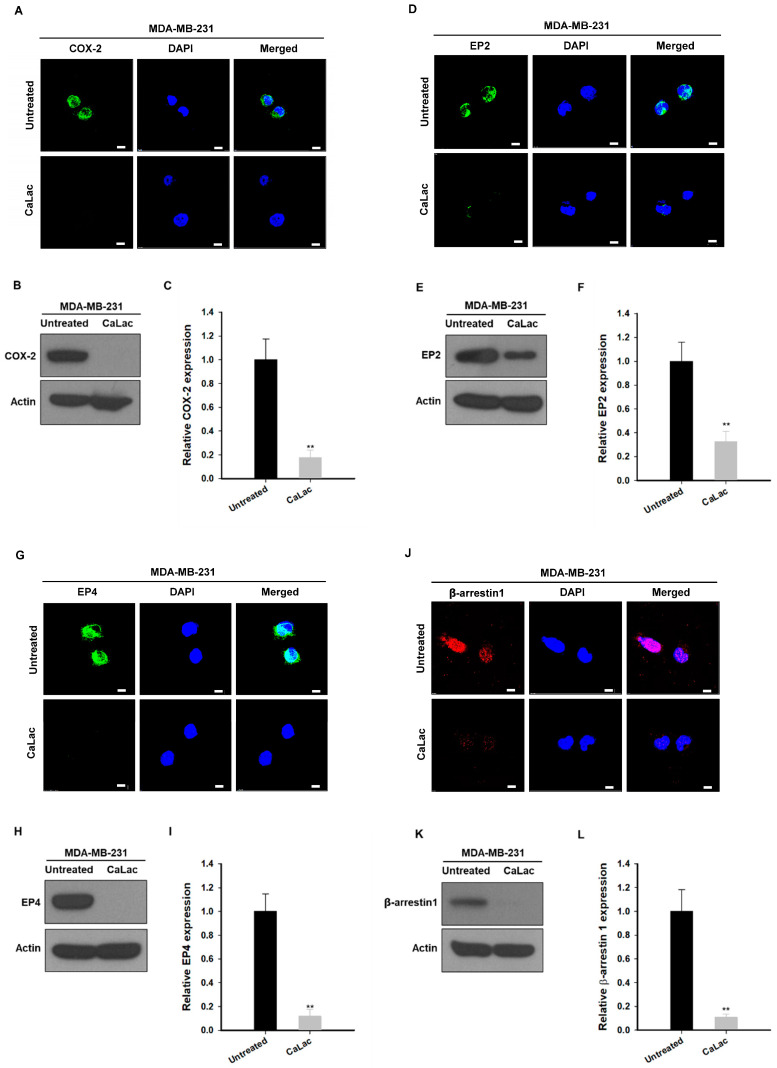
Inhibitory effect on factors under control by nuclear factor kappa B (NF-κB) associated with trans-activation of epidermal growth factor receptors (EGFRs) by calcium supply in triple-negative breast cancer cells. (**A**,**D**,**G**,**J**) Immunocytochemical analysis for the expression of cyclooxygenase-2 (COX-2), prostaglandin E2 receptor (EP) 2, EP4, or β-arrestin 1. Scale bars = 10 µm. (**B**,**E**,**H**,**K**) Western blot analysis for the expression of COX-2, EP2, EP4, or β-arrestin 1. The source films of Western blot data are provided in Supplementary Figure S3. (**C**,**F**,**I**,**L**) Quantitative analysis of the protein expression level of COX-2, EP2, EP4, or β-arrestin 1. A quantity of 5 mM lactate calcium salt (CaLac) was supplied in the CaLac group for 48 h. The bar graphs were made based on triplicate analysis. ** *p* < 0.001 vs. Untreated. Results are represented as means ± standard deviations.

Among the factors that were controlled by NF-κB and affected the survival of TNBC cells, we focused on the change in factors involved in EGFR trans-activation (Figure 3). The fluorescence images show that the intensity of cyclooxygenase-2 (COX-2; green fluorescence), EP2 (green fluorescence), EP4 (green fluorescence), and β-arrestin (red fluorescence) tended to decrease qualitatively following calcium supply (Figure 3A,D,G,J). In quantitative analysis of protein expression, COX-2 was significantly reduced to about 82.4% (Figure 3B,C), EP2 to about 77.2% (Figure 3E,F), EP4 to about 88.1% (Figure 3H,I), and β-arrestin to about 89.2% (Figure 3K,L) following calcium supply compared with the untreated group.

### 2.4. Calpeptin Reduces the Effect of Calcium Inhibiting Src-Dependent Trans-Activation of EGFR in TNBC Cells

**Figure 4:**
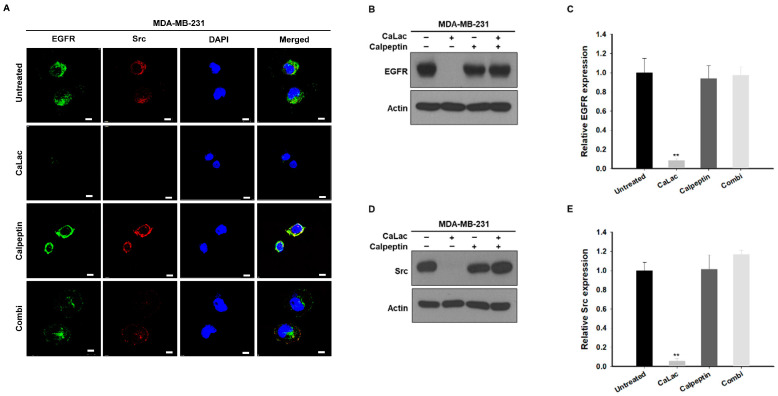
Investigation of whether calpeptin offsets the inhibitory effect of calcium on Src-mediated epithelial growth factor receptor (EGFR) trans-activation in triple-negative breast cancer cells. (**A**) Immunocytochemical analysis for the expression of EGFR and Src following co-treatment with 20 µM calpeptin. Scale bars = 10 µm. (**B**,**D**) Western blot analysis for the expression of EGFR or Src following co-treatment with 20 µM calpeptin. The source films of Western blot data are provided in Supplementary Figure S4. (**C**,**E**) Quantitative analysis of the protein expression level of EGFR or Src following co-treatment with 20 µM calpeptin. A quantity of 5 mM lactate calcium salt (CaLac) was supplied in the CaLac and Combi groups for 48 h. The bar graphs were made based on triplicate analysis. ** *p* < 0.001 vs. Untreated. Results are represented as means ± standard deviations.

Changes in the expression of EGFR and Src were investigated following treatment with calpeptin to confirm whether inhibition of Src-dependent trans-activation of EGFR was realized by sustained calcium supply (Figure 4). The fluorescence intensity of EGFR (green fluorescence) and Src (red fluorescence) was still maintained in the combi group (combination of calpeptin and CaLac) while it was decreased by sustained calcium supply (the CaLac group; Figure 4A). Quantitative analysis indicated that the inhibitory effect on EGFR or Src expression of the sustained calcium supply was offset following co-treatment with calpeptin (Figure 4B–E). There was no significant effect on EGFR or Src expression of calpeptin only (Figure 4).

### 2.5. Calpeptin Reduces the Effect of Calcium Inhibiting the Sub-Signals Stimulated by EGFR and Src in TNBC Cells

**Figure 5:**
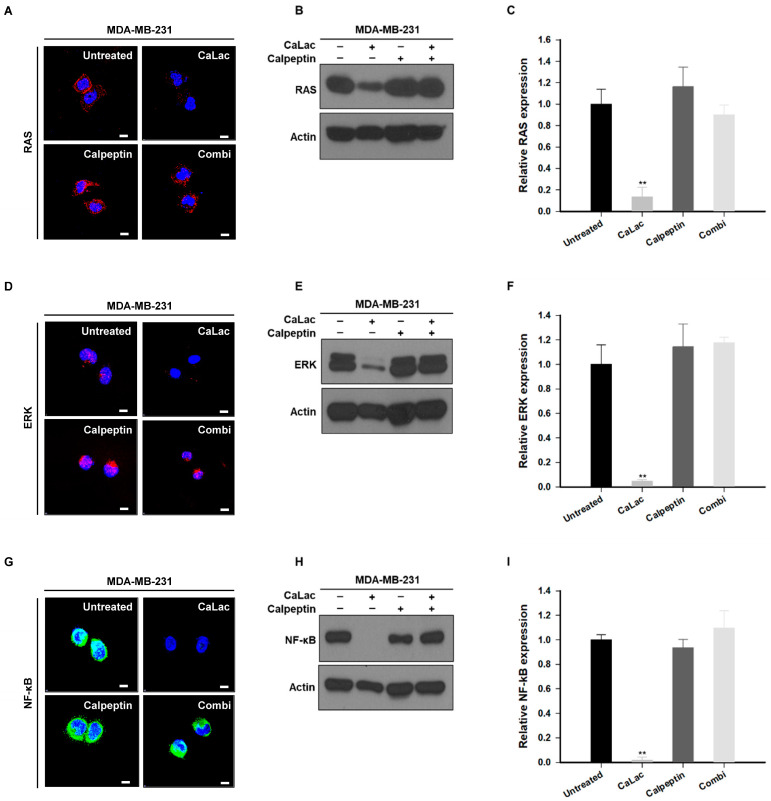
Investigation of whether calpeptin offsets the inhibitory effect of calcium on the sub-signaling of epithelial growth factor receptor (EGFR) and Src in triple-negative breast cancer cells. (**A**,**D**,**G**) Immunocytochemical analysis for the expression of RAS, ERK, or nuclear factor kappa B (NF-κB) following co-treatment with 20 µM calpeptin. Scale bars = 10 µm. Unmerged images are shown in Supplementary Figure S5. (**B**,**E**,**H**) Western blot analysis for the expression of RAS, ERK, or NF-κB following co-treatment with 20 µM calpeptin. The source films of Western blot data are provided in Supplementary Figure S6. (**C**,**F**,**I**) Quantitative analysis of the protein expression levels of RAS, ERK, or NF-κB following co-treatment with 20 µM calpeptin. A quantity of 5 mM lactate calcium salt (CaLac) was supplied in the CaLac and the combi groups for 48 h. The bar graphs were made based on triplicate analysis. ** *p* < 0.001 vs. Untreated. Results are represented as means ± standard deviations.

The effect on the expression of RAS, ERK, and NF-κB governed by the activity of EGFR and Src was investigated following single or co-treatment of CaLac and calpeptin. While fluorescence intensities of RAS, ERK (red fluorescence), and NF-κB (green fluorescence) were decreased by sustained calcium supply, the intensities were maintained as well as that of the control group by the combination with calpeptin (the combi group; Figure 5A,D,G). In the qualitative analysis of the protein blots, the expression of RAS, ERK, and NF-κB was reduced in the CaLac group, however, the expression was not decreased following the co-treatment of CaLac and calpeptin (Figure 5B,E,H). Quantitative analysis indicated that expression levels of the RAS, ERK, and NF-κB were significantly decreased by sustained calcium supply, and such an inhibitory effect was offset following co-treatment of CaLac and calpeptin (Figure 5C,F,I).

### 2.6. Calpeptin Reduces the Effect of Calcium Inhibiting the EP Stimulation Resulting from NF-κB Downregulation in TNBC Cells

**Figure 6:**
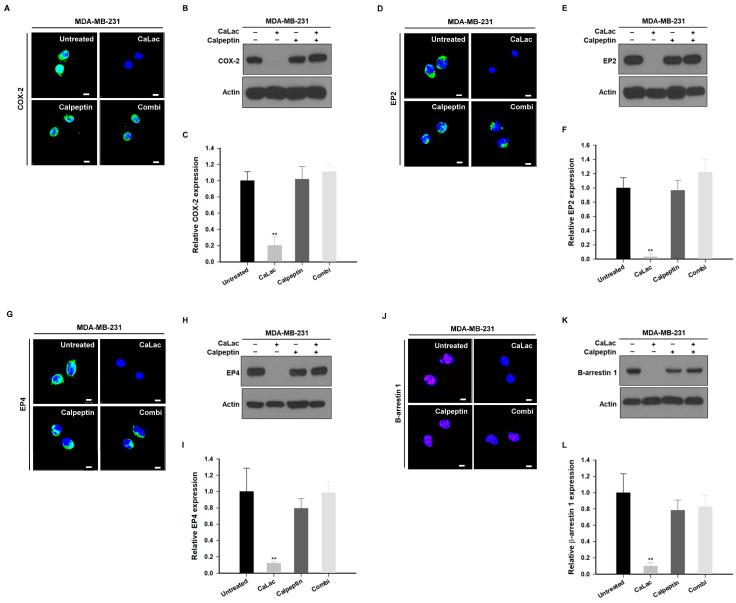
Investigation of whether calpeptin offsets the inhibitory effect of calcium on prostaglandin E2 receptor (EP) stimulation resulting from nuclear factor kappa B (NF-κB) downregulation in triple-negative breast cancer cells. (**A**,**D**,**G**,**J**) Immunocytochemical analysis of the expression of cyclooxygenase-2 (COX-2), EP2, EP4, and β-arrestin 1 following co-treatment with 20 µM calpeptin. Scale bars = 10 µm. Unmerged images are shown in Supplementary Figure S7. (**B**,**E**,**H**,**K**) Western blot analysis for the expression of COX-2, EP2, EP4, and β-arrestin 1 following co-treatment with 20 µM calpeptin. The source films of Western blot data are provided in Supplementary Figure S8. (**C**,**F**,**I**,**L**) Quantitative analysis for the protein expression level of COX-2, EP2, EP4, and β-arrestin 1 following co-treatment with 20 µM calpeptin. A quantity of 5 mM lactate calcium salt (CaLac) was supplied in the CaLac and the combi groups for 48 h. The bar graphs were made based on triplicate analysis. ** *p* < 0.001 vs. Untreated. Results are represented as means ± standard deviations.

The effect on the expression of EP stimulation factors governed by the activity of NF-κB was investigated following single or co-treatment of CaLac and calpeptin. While fluorescence intensities of COX-2, EP2, EP4 (green fluorescence), and β-arrestin 1 (red fluorescence) were decreased by sustained calcium supply, the intensities were maintained by the combination with calpeptin (the combi group) as well as that of the control group (Figure 6A,D,G,J). In the qualitative analysis of the protein blots, COX-2, EP2, EP4, and β-arrestin 1 levels were reduced in the CaLac group, while the expression of the EP stimulation factors was not decreased following the co-treatment of CaLac and calpeptin (Figure 6B,E,H,K). Quantitative analysis indicated that COX-2, EP2, EP4, and β-arrestin 1 expression levels were significantly decreased by sustained calcium supply, and such an inhibitory effect was decreased following co-treatment of CaLac and calpeptin (Figure 6C,F,I,L).

### 2.7. Sustained Calcium Supply Shows an Anticancer Effect on TNBC

**Figure 7:**
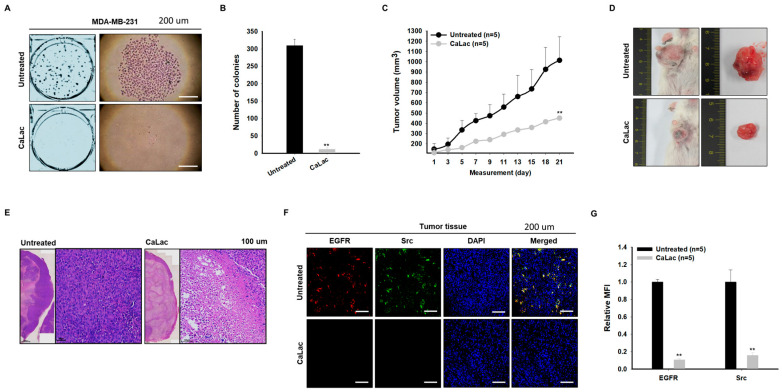
Investigation of anticancer effect of sustained calcium supply targeting triple-negative breast cancer (TNBC). (**A**,**B**) Comparison of clonogenic ability in TNBC cells. A quantity of 5 mM lactate calcium salt (CaLac) was supplied in the CaLac group for 48 h. Scale bars = 200 µm. (**C**) Comparison of tumor volume in the xenograft mice transplanted with TNBC cells. A quantity of 20 mg/kg CaLac was subcutaneously administered in the CaLac group for 21 days daily. (**D**) Representative pictures of tumor mass after autopsy. (**E**) H&E staining to compare the degree of tumor tissue cell death. Scale bars = 100 µm. (**F**) Immunocytochemical analysis for the expression of epithelial growth factor receptor (EGFR) and Src in tumor tissues. Scale bars = 200 µm. (**G**) Quantitative analysis for immunocytochemical expression of EGFR and Src by mean fluorescence intensity (MFI). A total of 1 × 10^3^ cells was analyzed in each case. ** *p* < 0.001 vs. Untreated. Results are represented as means ± standard deviations.

The clonogenic ability of TNBC cells was suppressed by sustained calcium supply; it was observed that the colony number was 309.3 ± 18.37 and 12 ± 4.78 in the control and the CaLac group, respectively (Figure 7A,B). When tumor growth was observed in the xenograft mice transplanted with TNBC, tumor volume was decreased by about 56% following sustained calcium supply compared with the untreated group (Figure 7C,D). While the intact nucleus of the tumor tissue cells was observed in the untreated group, apoptotic nucleus condensation was observed in most areas of the tumor treated with CaLac even if the central necrosis of the harvested tumor was excluded (Figure 7E). The fluorescence reaction of EGFR (red fluorescence) and Src (green fluorescence) in tumor tissues was decreased qualitatively following sustained calcium supply (Figure 7F). In quantitative analysis of the fluorescence intensities, EGFR was significantly reduced to about 84.3% and Src to about 89.4% in the CaLac group compared with the untreated group (Figure 7G).

### 2.8. Sustained Calcium Supply Inhibits the Sub-Signals to Be Stimulated from EGFR and Src in TNBC Tissue

**Figure 8:**
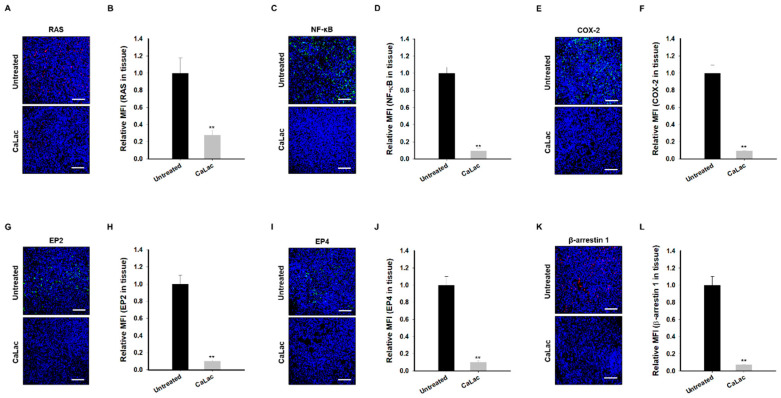
Inhibitory effect on sub-signaling of epidermal growth factor receptor (EGFR) and Src and EP-stimulating factors due to calcium supply in triple-negative breast cancer tissue. (**A**,**C**,**E**,**G**,**I**,**K**) Immunofluorescence analysis in tumor sections for the expression of RAS, nuclear factor kappa B (NF-κB), cyclooxygenase-2 (COX-2), prostaglandin E2 receptor (EP) 2, EP4, and β-arrestin 1. Scale bars = 200 µm. Unmerged images are shown in Supplementary Figure S9. (**B**,**D**,**F**,**H**,**J**,**L**) Quantitative analysis for immunofluorescence of RAS, NF-κB, COX-2, EP2, EP4, and β-arrestin 1 by mean fluorescence intensity (MFI). A total of 1 × 10^3^ cells was analyzed in each case. ** *p* < 0.001 vs. Untreated. Results are represented as means ± standard deviations.

The inhibitory effect of sub-signals and EP-stimulating factors to be stimulated by EGFR and Src was observed in tumor tissues. Downregulation of RAS (red fluorescence; Figure 8A) led to suppression of NF-κB expression (green fluorescence; Figure 8C), followed by the decrease in the expression of EP-stimulating factors, COX-2 (green fluorescence; Figure 8E), EP2 (green fluorescence; Figure 8G), EP4 (green fluorescence; Figure 8I), and β-arrestin 1 (red fluorescence; Figure 8K). In quantitative analysis of the fluorescence intensities, RAS, NF-κB, and EP-stimulating factors were significantly reduced in the CaLac group by about 72.4% (RAS; Figure 8B), 90.4% (NF-κB; Figure 8D), 90.6% (COX-2; Figure 8F), 90.1% (EP2; Figure 8H), 90.2% (EP4; Figure 8J), and 92.9% (β-arrestin 1; Figure 8L) compared with the untreated group.

## 3. Discussion

This study investigated the possibility of inducing an anticancer effect by calcium-mediated Src degradation. EGFR trans-activation was suppressed following Src degradation, and then the sub-signals of EGFR, such as RAS, ERK, and NF-ĸB, and EP-stimulating factors, were downregulated. It could be that the induced anticancer effect targeting TNBC was a result of this mechanism. As aforementioned, TNBC is defined by the lack of expression of ER, PR, and HER2; therefore, the EGFR is frequently overexpressed [2,7]. As is well known, EGFR is a proto-oncogene that enhances cancer cell proliferation and survival [7,10]. Recent studies have revealed that the ubiquitin domain, such as ubiquitin-specific proteases, and Src are overexpressed in TNBC, so they are involved in the regulation of EGFR signaling to support malignant growth, invasion, and metastasis [14,15]. Among the factors involved in the survival pathway of EGFR, the function of Src has rarely been studied in the mainstream, but attempts have been made to establish a treatment for TNBC by targeting Src as a druggable target. Src is known as the activator of EGFR downstream signaling in cancer cells as a signaling transporter inducing phosphorylation of EGFR and causes the activation of the signal-regulating kinases, such as RAS and ERK [11,16]. More specifically, the activation of EGFR signaling mediated by Src promotes RAS activation, one of the essential signals for cancer cell growth, differentiation, and survival, and consequently induces the upregulation of NF-κB [14,15,16]. Since NF-κB acts as a transcriptional regulator, it plays a critical role as a main factor in the expression of COX-2; then, the increase in COX-2 mediates the biogenesis of prostaglandin E2 (PGE2) [17,18]. The increased PGE2 induced in this process acts as a ligand that activates four subtypes of PEG2 receptor (EP1-4), and this process leads to a malignant cycle that further induces EGFR signal transduction triggered by Src and contributes to the development of TNBC [18,19,20]. In the present study, it was possible to inhibit all of the processes in this series by inducing a decrease in Src by sustained calcium supply, which eventually interfered with the malignant cycle of survival signals in TNBC derived from the EGFR signals, resulting in inducing an anticancer effect. Further, it has been found that the decrease in Src, a key factor for the anticancer effect targeting TNBC, was due to the action of calpain, which was induced by an increase in intracellular calcium by CaLac. Previous studies have reported the results of investigating the role of calpain in inhibiting the kinase function of Src to EGFR [13,21]. In this study, the inhibitory effect of EGFR and its downstream signaling by calcium supply in TNBC was abolished by calpeptin, a calpain inhibitor [22]. The critical role of calpain in regulating Src activation can be emphasized, as in previous studies, by deriving a result that offsets the effect of calcium supply [13]. In this study, a high dose of calcium was used in the in vitro study because of homeostasis to maintain the optimal calcium concentration in the cells [23]. Since the low concentration of calcium is rapidly excreted out of the cells, it is considered that the accumulation of intracellular calcium in TNBC cells could be induced only by a high-concentration calcium supply. Given these conditions, our research team does not believe that the effects of high doses of intracellular calcium contribute only to the activation of calpain. Therefore, follow-up studies on a range of factors related to survival threats of TNBC cells, such as increased reactive oxygen species, will be conducted in the future.

## 4. Materials and Methods

### 4.1. Cell Culture and Reagent

Triple-negative breast cancer cells (MDA-MB-231) were obtained from the American Type Culture Collection (ATCC, Manassas, VA, USA). MDA-MB-231 cells were cultured in RPMI-1640 medium (about 0.000524 mM calcium included; Welgene, Daegu, Republic of Korea) supplemented with 10% fetal bovine serum (Welgene) and 1% penicillin/streptomycin (Welgene) at 37 °C in 5% CO_2_. CaLac was purchased from Sigma-Aldrich (St. Louis, MO, USA) and dissolved in distilled water. The solution was stored at 4 °C until use.

### 4.2. Cell Viability Assay

MDA-MB-231 cells were cultured in a 96-well plate for 24 h at an initial density of 3 × 10^3^ cells/well, before treatment with different concentrations (1.0, 2.5, and 5.0 mM) of CaLac for 24, 48, or 72 h at 37 °C. After CaLac treatment, 10 μL of 3-(4,5-dimethylthiazol-2-yl)-2,5-diphenyltetrazolium bromide was added to each well, and then the cells were incubated for 1 h at 37 °C in a humidified environment of 5% CO_2_. After discarding the media, 200 μL of dimethyl sulfoxide (Cell Signaling Technology, Danvers, MA, USA) was added to each well. The absorbance was then read at 570 nm using a microplate reader (iMark Microplate Absorbance Reader, Bio-Rad, Hercules, CA, USA).

### 4.3. Immunocytochemistry

MDA-MB-231 cells were seeded onto a bio-coated coverslip (BD Bioscience, San Jose, CA, USA) at a density of 1.0 × 10^5^ cells. After 24 h of 2.5 mM CaLac treatment, the cells were fixed using 4% paraformaldehyde. Subsequently, the coverslips were incubated with the primary antibodies for 15 h at 4 °C. The primary antibodies used were as follows: Src (1:300, Santa Cruz Biotechnology, Santa Monica, CA, USA); EGFR (1:300, Cell Signaling); RAS (1:300, Santa Cruz Biotechnology); ERK (1:300, Cell Signaling); NF-κB (1:300, Santa Cruz Biotechnology); COX-2 (1:300, Santa Cruz Biotechnology); EP2 (1:200, Cayman Chemical Company, Ann Arbor, MI, USA); EP4 (1:200, Santa Cruz Biotechnology); and β-Arrestin 1 (1:300, Abcam, Cambridge, MA, USA). After PBS wash, the cells were incubated with anti-rabbit secondary biotinylated antibody (1:2000, Vector Laboratories, Burlingame, CA, USA) and visualized using streptavidin conjugated to fluorescein (Vector Laboratories). For double immunostaining, the cells were incubated with anti-mouse secondary biotinylated antibody (1:2000, Vector Laboratories) and visualized using streptavidin conjugated to fluorescein (Vector Laboratories). The coverslips were mounted on microscope slides with VECTASHIELD^®^ Hard Set™ mounting medium with 4′,6-diamidino-2-phenylindole (Vector Laboratories). Fluorescence images were obtained by confocal laser scanning microscopy (LSCM, Nikon A1+, Tokyo, Japan).

### 4.4. Protein Extraction

MDA-MB-231 cells were seeded in a 6-well plate (3.0 × 10^3^ cells/well) and treated with 2.5 mM CaLac for 6 h. After incubation, the cells were washed twice with ice-cold PBS, and lysed with a protein lysis buffer (1% NP-40, 0.25% sodium deoxycholate, 150 mM NaCl, 1 mM EDTA, 1% Triton X-100, 50 mM Tris-HCl (pH 7.4), 10% glycerol) with protease inhibitor cocktail (Roche, Basel, Switzerland) and phosphatase inhibitors (Na_3_VO_4_ 1 mM, NaF 100 mM, NaPP 10 mM) for 30 min at 4 °C. The lysates were centrifuged at 12,000× *g* for 20 min at 4 °C. The supernatant was used for Western blot analysis.

### 4.5. Western Blot Analysis

Proteins were separated on 8% sodium dodecyl sulfate-polyacrylamide gels and transferred onto polyvinylidene difluoride membranes. Then, the membranes were blocked in 5% non-fat milk (Bio-Rad) for 1 h at room temperature and were incubated overnight at 4 °C with primary antibody diluted in Tris-buffered saline containing Tween 20 (TBST), 5% bovine serum albumin, and 0.1% sodium azide (Sigma-Aldrich). The specific primary antibodies used were as follows: Actin (1:1000, Santa Cruz Biotechnology); Src (1:1000, Cell Signaling); RAS (1:1000, Santa Cruz Biotechnology); ERK (1:1000, Cell Signaling); NF-κB (1:1000, Santa Cruz Biotechnology); EGFR (1:1,000, Cell Signaling); COX-2 (1:1000, Santa Cruz Biotechnology); EP2 (1:500, Cayman Chemical Company); EP4 (1:500, Cayman Chemical Company); and phospho-β-Arrestin 1 (1:1000, Abcam). The membranes were washed with TBST and then incubated with secondary antibodies at room temperature for 2 h. The secondary antibodies used were as follows: anti-rabbit secondary antibody (1:10,000, Abclone, Seoul, Republic of Korea) and anti-mouse secondary antibody (1:10,000, Abclone). The membranes were exposed to X-ray film (Agfa, Leverkusen, Germany) according to the manufacturer’s protocol.

### 4.6. Clonogenic Assay

MDA-MB-231 cells were seeded at a density of 3 × 10^2^ cells/well in a 6-well plate and incubated for 24 h at 37 °C. After 24 h, the medium was removed, and the cells were treated with CaLac for 14 days in a 5% CO_2_-humidified atmosphere at 37 °C. After 14 days, the colonies were fixed with methanol and stained with hematoxylin (Thermo Fisher Scientific, Waltham, MA, USA). The colonies were counted under an optical microscope (Olympus, Center Valley, PA, USA).

### 4.7. Xenograft Animal Model

All experiments were performed under the institutional guidelines established by the Institutional Animal Care and Use Committee of Gachon University (IACUC-LCDI-2019-0102, 16 July 2019). Five-week-old female immunodeficiency (NOD-SCID) mice were purchased from Samtako Bio Korea (Osan, Gyeonggi-do, Republic of Korea). All animals were maintained in a 12 h light/dark cycle (light on, 08:00 h) at 22 to 25 °C, with free access to food and water. MDA-MB-231 cells (1 × 10^7^) were inoculated into the subareolar area of the mice. When the tumor grew to about 150 mm^3^, 20 mg/kg of CaLac was subcutaneously injected into the interscapular region for 21 days. Tumor size was determined three times per week using digital calipers, and the tumor volume was calculated using the following formula: V = (L × W^2^)/2 (L, length; W, width). At the end of the experiment, all tumors were harvested and used for histological analysis.

### 4.8. Immunofluorescence

The tumor tissues were fixed with 10% neutral buffered formalin and embedded in paraffin for sectioning. Tissue sections (5 µm thick) were made from the paraffin-embedded block using a microtome. The sectioned tissues were incubated in ethanol series (100%, 95%, 70%, and 50%) for deparaffinization, and then endogenous peroxidase activity was blocked with 3% hydrogen peroxide in distilled water for 30 min. Heat-induced antigen retrieval was performed in 10 mM citrate buffer (pH 6.0) in a microwave oven, followed by washing with PBS. The sections were blocked using a blocking agent (Invitrogen, Frederick, MD, USA) and incubated overnight with the primary antibodies at 4 °C. The primary antibodies used were as follows: EGFR (1:50, Cell Signaling); Src (1:50, Santa Cruz Biotechnology); RAS (1:50, Santa Cruz Biotechnology); NF-κB (1:50, Santa Cruz Biotechnology); COX-2 (1:50, Santa Cruz Biotechnology); EP2 (1:50, Cayman Chemical Company); EP4 (1:50, Santa Cruz Biotechnology); and β-Arrestin 1 (1:100, Abcam). After PBS wash, the cells were incubated with an anti-rabbit secondary biotinylated antibody (1:200, Vector Laboratories) and visualized using streptavidin conjugated to fluorescein (Vector Laboratories). For double immunostaining, the cells were incubated with anti-mouse secondary biotinylated antibody (1:200, Vector Laboratories) and visualized using streptavidin conjugated to fluorescein (Vector Laboratories). The coverslips were mounted on microscope slides with VECTASHIELD^®^ Hard Set™ mounting medium with 4′,6-diamidino-2-phenylindole (Vector Laboratories). Fluorescence images were obtained by confocal laser scanning microscopy (LSCM, Nikon A1).

### 4.9. Statistical Analysis

All data have been presented as means ± standard deviations. Significance was analyzed using the Student’s t-test or one-way analysis of variance (ANOVA), based on the normality of the data. *p* < 0.05 was considered significant. All statistical analyses were performed using Sigma Stat (ver. 3.5, Systat Software Inc., San Jose, CA, USA).

## 5. Conclusions

This study demonstrated the possibility of an anticancer effect on TNBC following Src degradation mediated by calcium-dependent calpain followed by suppression of the downstream signaling of EGFR. Since this study is still in the non-clinical early stage, an advanced in vivo study is required to determine the response to toxicity caused by high doses of calcium. Further, exploring the form of an appropriate formulation that can be used in clinic or determining the optimal usage and dosage will be needed because calcium is an endogenous material that undergoes rapid metabolism.

## Data Availability

The data presented in this study are available on request from the corresponding authors.

## Supplementary Materials

The following supporting information can be downloaded at: https://www.mdpi.com/article/10.3390/ijms241713291/s1.
