# Supplementary material for: Suppressing Src-Mediated EGFR Signaling by Sustained Calcium Supply Targeting Triple-Negative Breast Cancer
Source: Int J Mol Sci. 2023 Aug 27;24(17):13291. doi: 10.3390/ijms241713291 (PMC10488068; doi:10.3390/ijms241713291)
Supplement: Supplementary file 1 [file ijms-24-13291-s001.zip › ijms-2560448-supplementary.pdf]

Supplementary Materials for

Suppressing Src-mediated EGFR signaling by sustained calcium supply targeting triple-negative breast cancer cells

Keun-Yeong Jeong [1,†,*], Seon Young Park [1,†], Min Hee Park [1], and Hwan Mook Kim[2,*]

*Correspondence to:

Keun-Yeong Jeong (alvirus@naver.com)

Hwan Mook Kim (painout@gmail.com)

This file includes:

Supplementary Figures. S1 to S9

Supplementary Figure S1

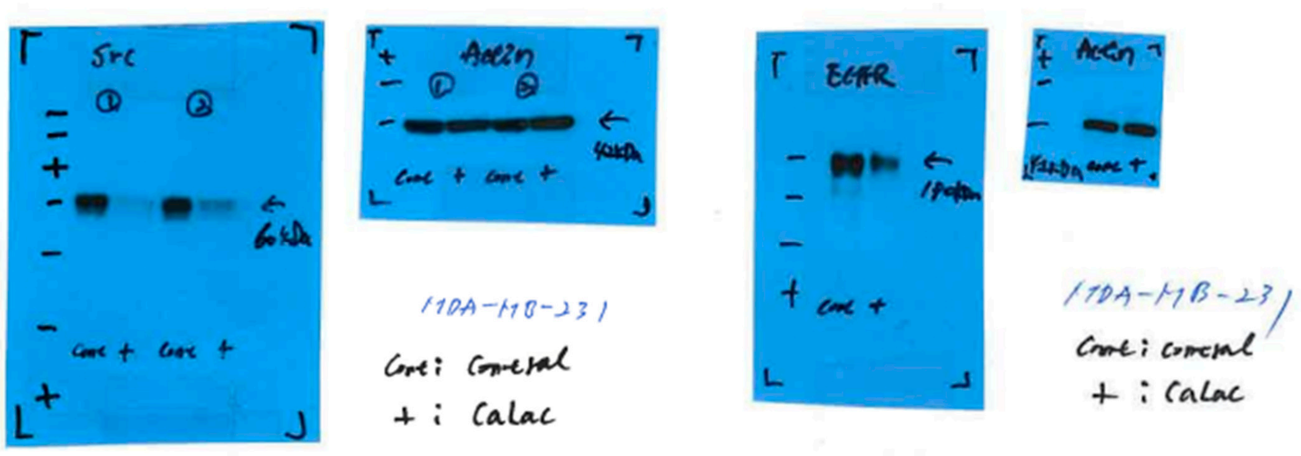

Figure S1. The source films of western blot data for Figures 1f and h.

Supplementary Figure S2

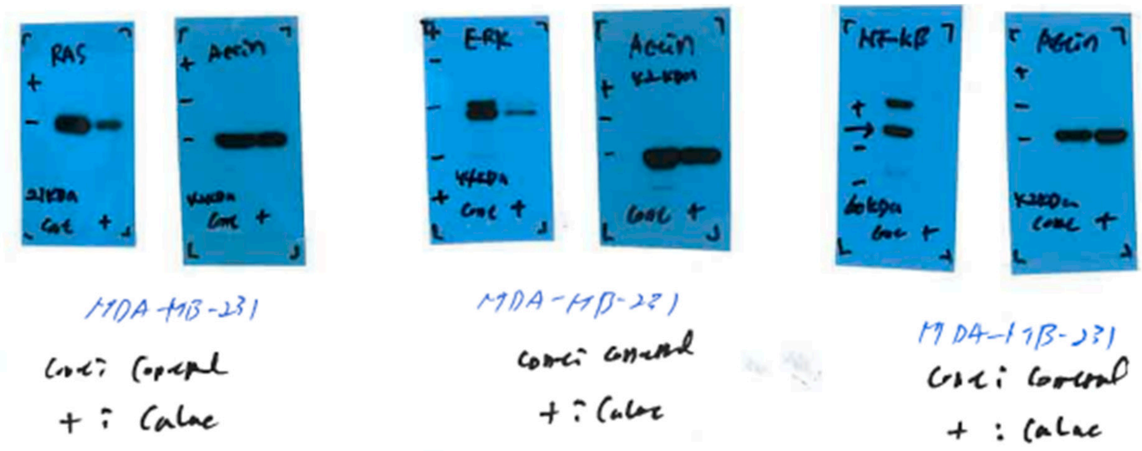

Figure S2. The source films of western blot data for Figures 2b, e, and h.

Supplementary Figure S3

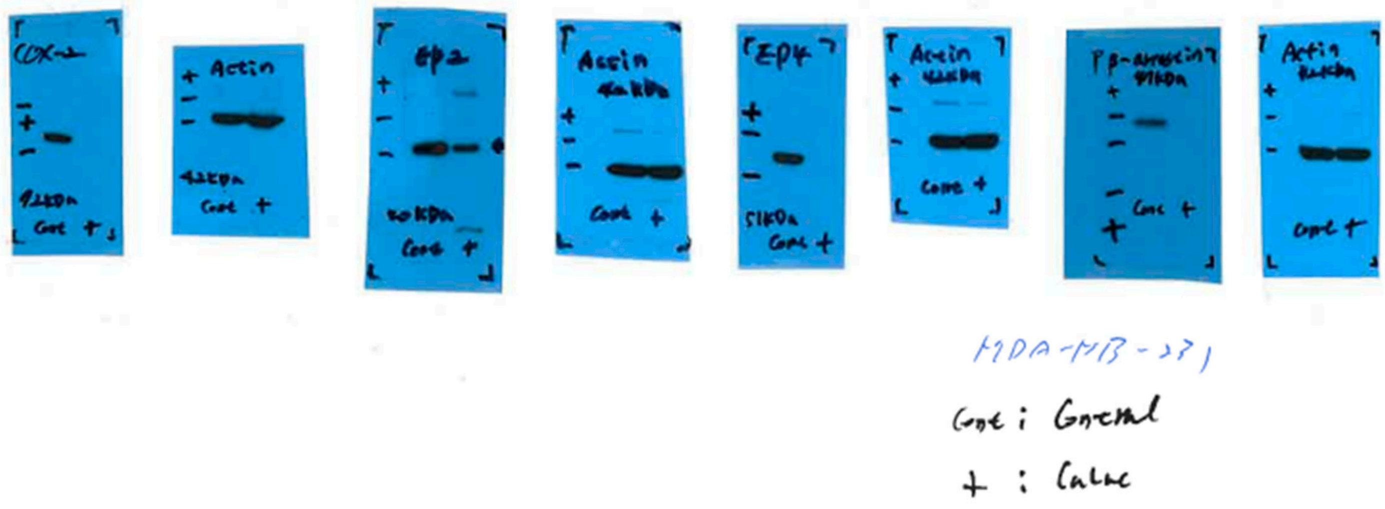

Figure S3. The source films of western blot data for Figures 3b, e, h, and k.

Supplementary Figure S4

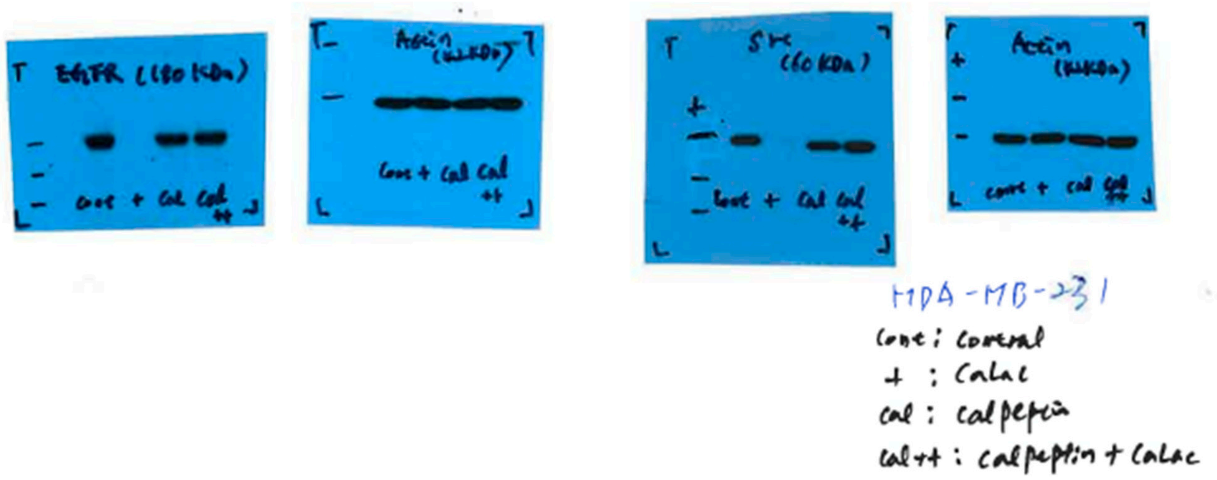

Figure S4. The source films of western blot data for Figures 4b and d.

Supplementary Figure S5

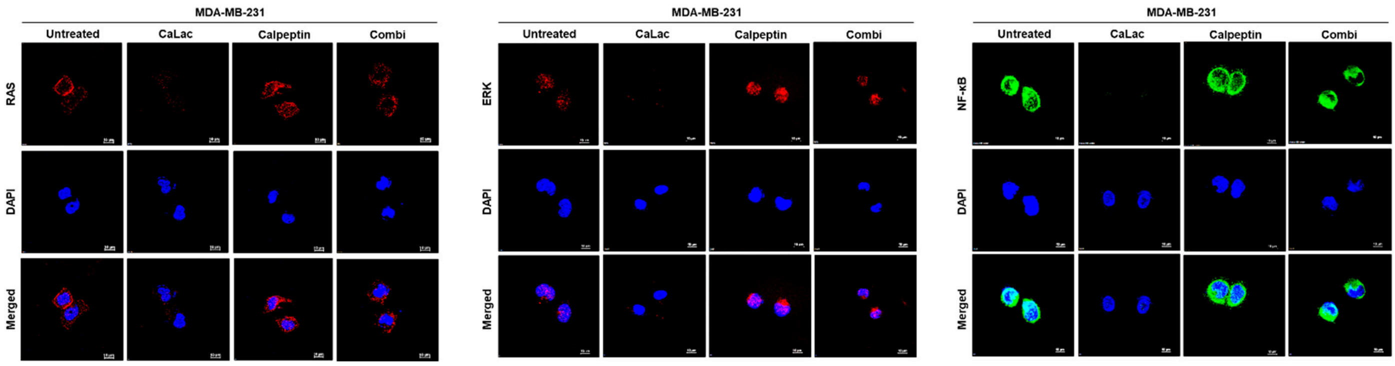

Figure S5. Unmerged immunocytochemical images for Figures 5a, d, and g. Scale bars = 10 μm.

Supplementary Figure S6

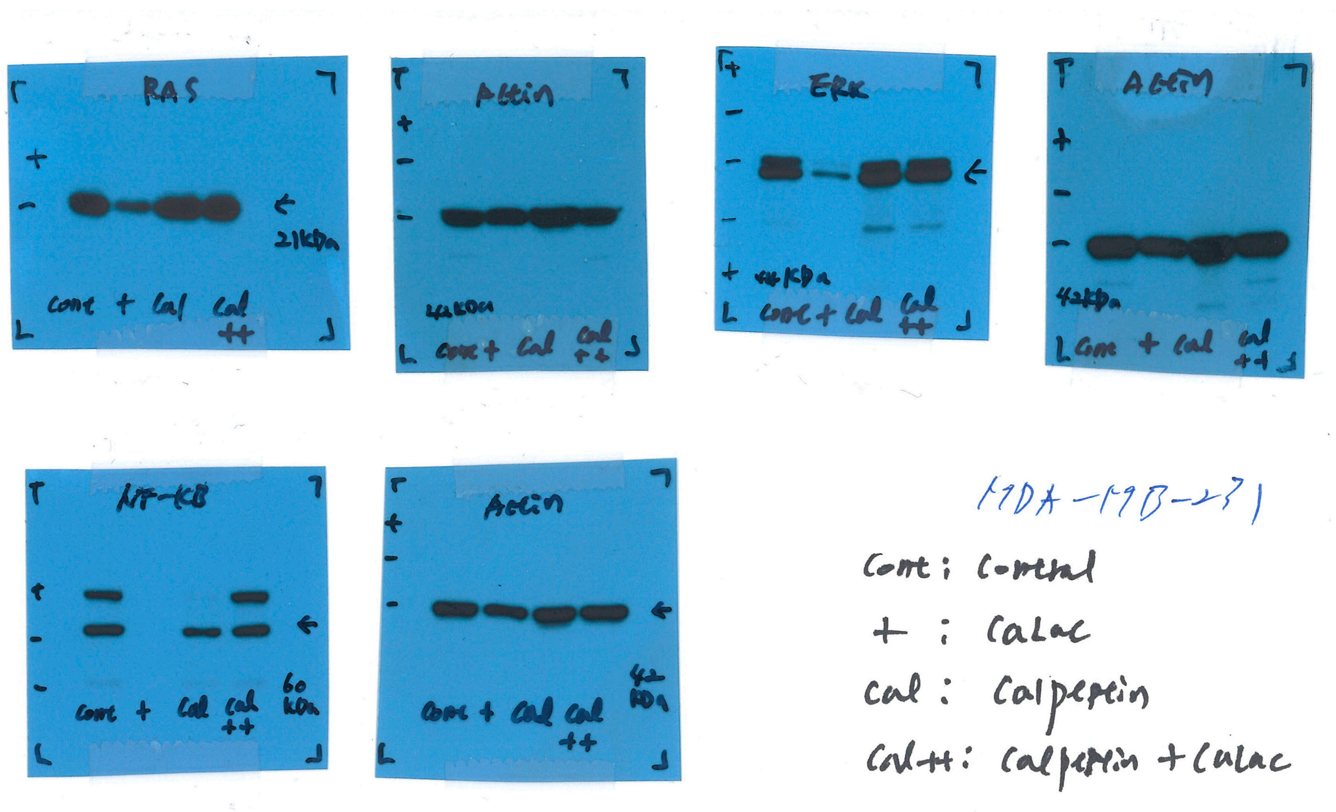

Figure S6. The source films of western blot data for Figures 5b, e, and h.

Supplementary Figure S7

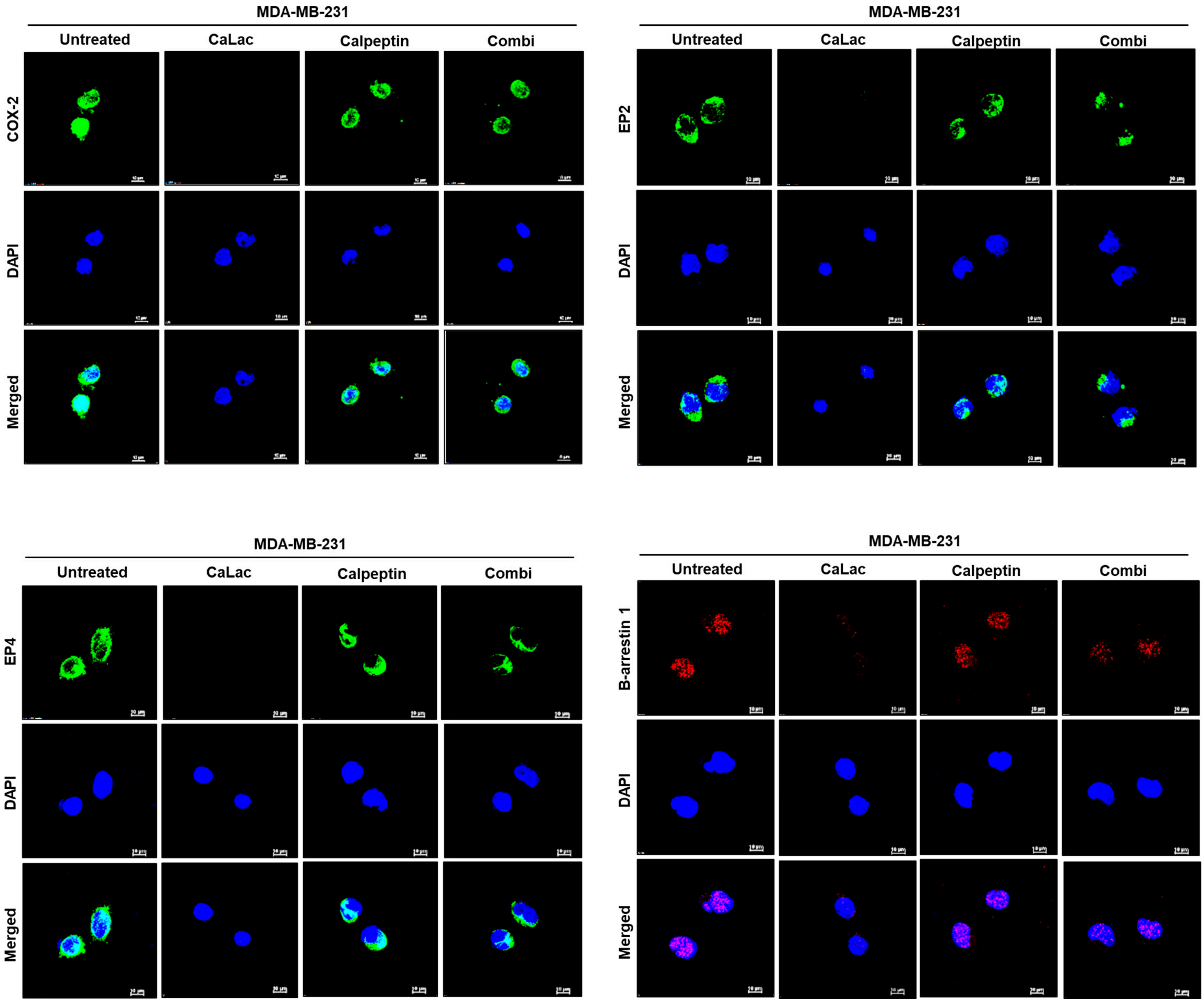

Figure S7. Unmerged immunocytochemical images for Figures 6a, d, g, and j. Scale bars = 10 μm.

Supplementary Figure S8

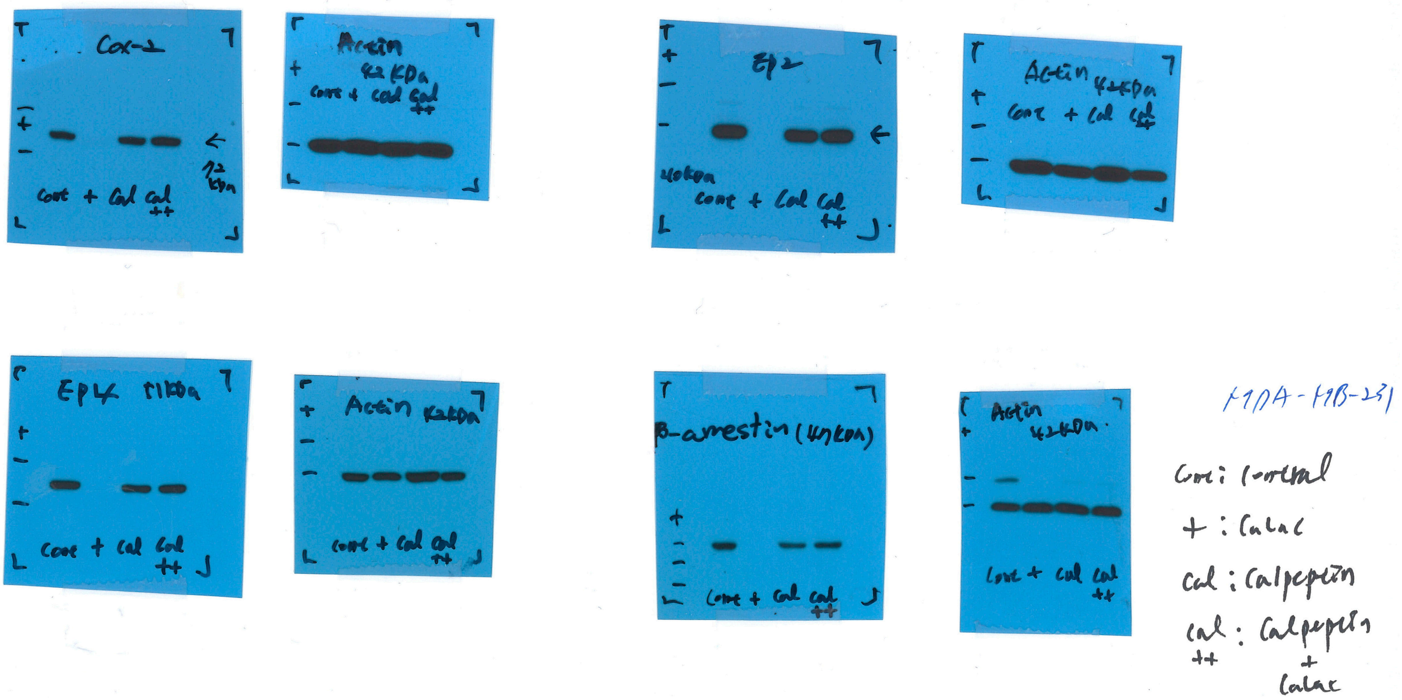

Figure S8. The source films of western blot data for Figures 6b, e, h, and k.

Supplementary Figure S9

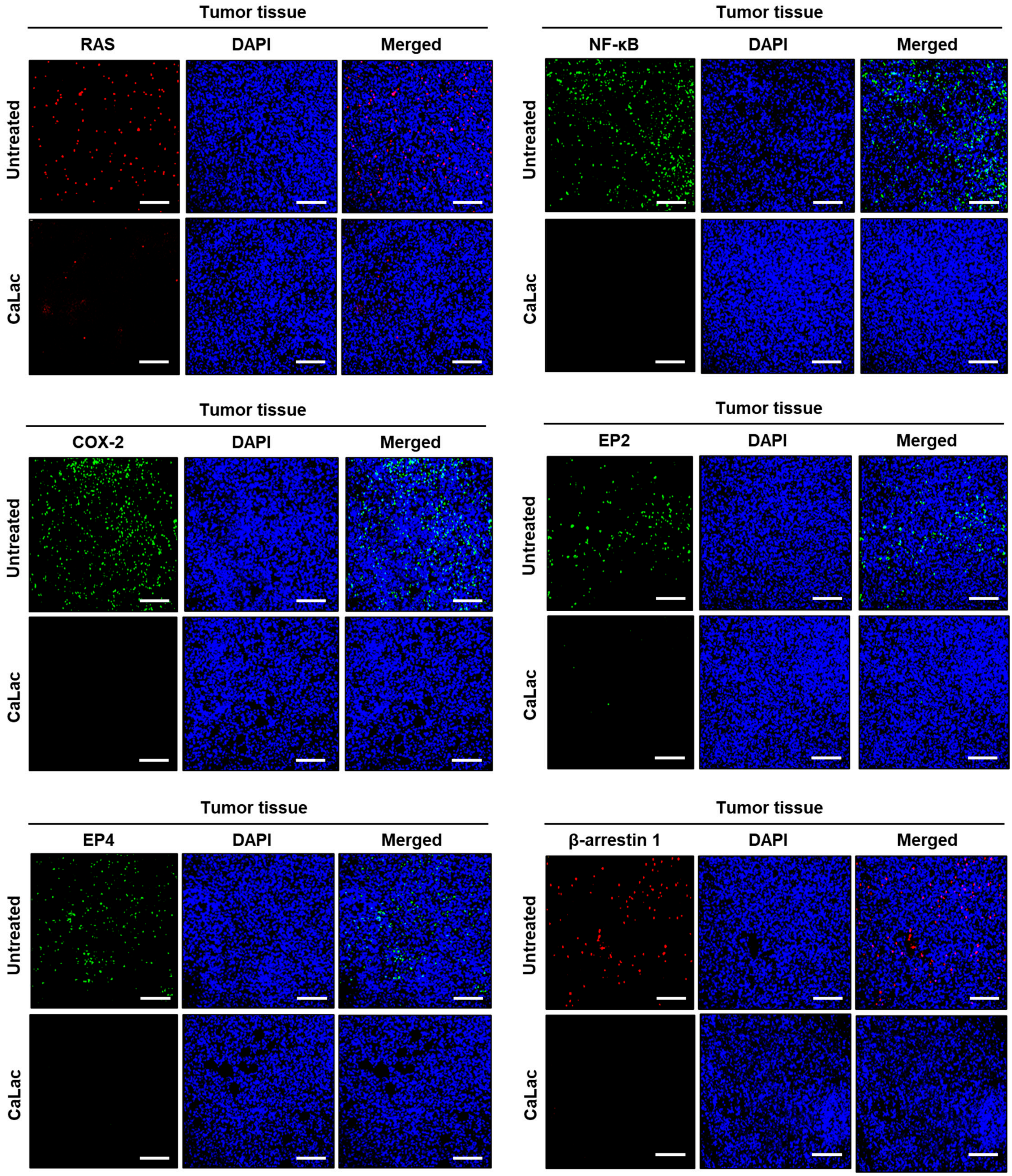

Figure S9. Unmerged immunofluorescence images of tumor tissues for Figures 8 a, c, e, g, i, and k. Scale bars = 200 μm.
